# Development of a novel high-entropy alloy with eminent efficiency of degrading azo dye solutions

**DOI:** 10.1038/srep34213

**Published:** 2016-09-28

**Authors:** Z. Y. Lv, X. J. Liu, B. Jia, H. Wang, Y. Wu, Z. P. Lu

**Affiliations:** 1 State Key Laboratory for Advanced Metals and Materials, University of Science and Technology Beijing, Beijing 100083, P. R. China

## Abstract

In addition to its scientific importance, the degradation of azo dyes is of practical significance from the perspective of environmental protection. Although encouraging progress has been made on developing degradation approaches and materials, it is still challenging to fully resolve this long-standing problem. Herein, we report that high entropy alloys, which have been emerging as a new class of metallic materials in the last decade, have excellent performance in degradation of azo dyes. In particular, the newly developed AlCoCrTiZn high-entropy alloy synthesized by mechanical alloying exhibits a prominent efficiency in degradation of the azo dye (Direct Blue 6: DB6), as high as that of the best metallic glass reported so far. The newly developed AlCoCrTiZn HEA powder has low activation energy barrier, i.e., 30 kJ/mol, for the degrading reaction and thus make the occurrence of reaction easier as compared with other materials such as the glassy Fe-based powders. The excellent capability of our high-entropy alloys in degrading azo dye is attributed to their unique atomic structure with severe lattice distortion, chemical composition effect, residual stress and high specific surface area. Our findings have important implications in developing novel high-entropy alloys for functional applications as catalyst materials.

Azo dyes, a kind of synthetic dyestuff, have wide applications in textile, printing, leather, food, and cosmetic industries due to their fast kinetics. The major structure of these dyes is characterized by the nitrogen to nitrogen double bonds “-N=N-”, which is fairly stable and recalcitrant to degrading^1^. As a consequence, the widespread applications of azo dyes are accompanied inevitably by environmental problems^2^,^3^,^4^. For example, the azo dye remnants in textile effluents are harmful to the aquatic ecological environment because they do prevent light penetration into water^5^. Therefore, it is of importance to degrade azo dyes existing in industrial wastewater.

Over the years, considerable efforts have been devoted to degrading azo dyes effluents, and as a consequence, various approaches have been developed to address this issue, such as physical absorption^6^,^7^, biological degradation^2^,^3^,^8^,^9^, photocatalytic degradation^1^,^10^,^11^,^12^ and degradation by zero-valent metals^13^,^14^,^15^,^16^. Nevertheless, most of them have their own limitations to some extent to fully resolve the long-standing problem. For instance, biological degradation is difficult to be controlled to reach a satisfactory level, especially in some complex environment containing various chemicals^9^, whilst light-degradation has limitations in transferring to commercial scale^15^. Currently, zero-valent metals have been extensively exploited in industrial applications because of their low cost, high degradation capability and simple operations, but their efficiency decays dramatically as a result of their rapid corrosion behavior in water, such as zero-valent-Mg^17^. Although the chemical stability and activity of zero-valent-metals can be improved by alloying with noble metals^18^, their commercialization is still challenging because of the high cost. Recently, it was reported that metallic glasses showed excellent capability in degrading azo dyes due to their thermodynamically metastable feature of atomic structures^17^,^19^,^20^,^21^. Unfortunately, synthesis of metallic glasses requires strict conditions, such as high purity of raw materials, rapid cooling, and high vacuum. Clearly, it is imperative to develop new materials which should have high degradation efficiency, low cost and can be easily prepared.

Recently, high-entropy alloys (HEAs) are emerging as a new class of metallic materials. Distinct from conventional alloys based mostly on one principal elements, HEAs are generally designed to contain at least five principal elements with equal or near-equal atomic percent^22^,^23^. The multi-principal elements feature leads to high mixing entropy and facilitates the formation of simple solid solution phases such as face-centered cubic (FCC) and/or body-centered cubic (BCC)^24^ and/or hexagonal closed-packed (HCP)^25^ which has been reported recently. In addition, due to different atomic sizes of constituents, the lattice of simply structured HEAs usually is severely distorted. These intrinsic features render HEAs unique properties^26^, such as high hardness^27^,^28^, high strength^29^, extraordinary fracture toughness^30^, and excellent corrosion resistance^31^,^32^. Therefore, HEAs have attracted extensive attention since their inception and become one of the research frontiers in the metallic materials field. Current researches on HEAs were mainly focused on mechanical properties, while functional properties, in particular chemical and catalytic properties, were seldom explored. It is interesting to notice that the solid-solution phase in HEAs are indeed firstly formed upon solidification, which are then kept to the ambient temperature due to the sluggish diffusion kinetics of HEAs, in a way analogous to the amorphous phase formation via the rapid solidification route^33^. So, in a way like metallic glasses^34^, HEAs are metastable thermodynamically compared to the ordinary crystalline alloys for the atoms residing on the high energy state as a result of the severe lattice distortion^33^,^35^. In view of the good chemical and catalytic properties of glassy alloys^36^,^37^, it is expected that the metastable HEAs could also possess excellent properties in degrading contaminants. Unfortunately, up to date, there is no any report about HEAs in this regard yet. Herein, we report degradation performances of three HEAs synthesized by mechanical alloying, i.e., AlCoCrTiZn (Sample1, denoted as S1), AlCoCrFeNi (Sample2, denoted as S2), and CoCrFeMnNi (Sample3, denoted as S3). The investigation demonstrates that the HEAs powders have higher reaction efficiency in degrading azo dyes than commercial zero-valent ion powders. In particular, the newly developed AlCoCrTiZn HEA powder exhibits an excellent performance, which is comparable to the best performance reported in metallic glasses so far. Our findings certainly will extend applications of HEAs as functional materials.

## Results

### Characterization of HEA powders

**Figure 1:**
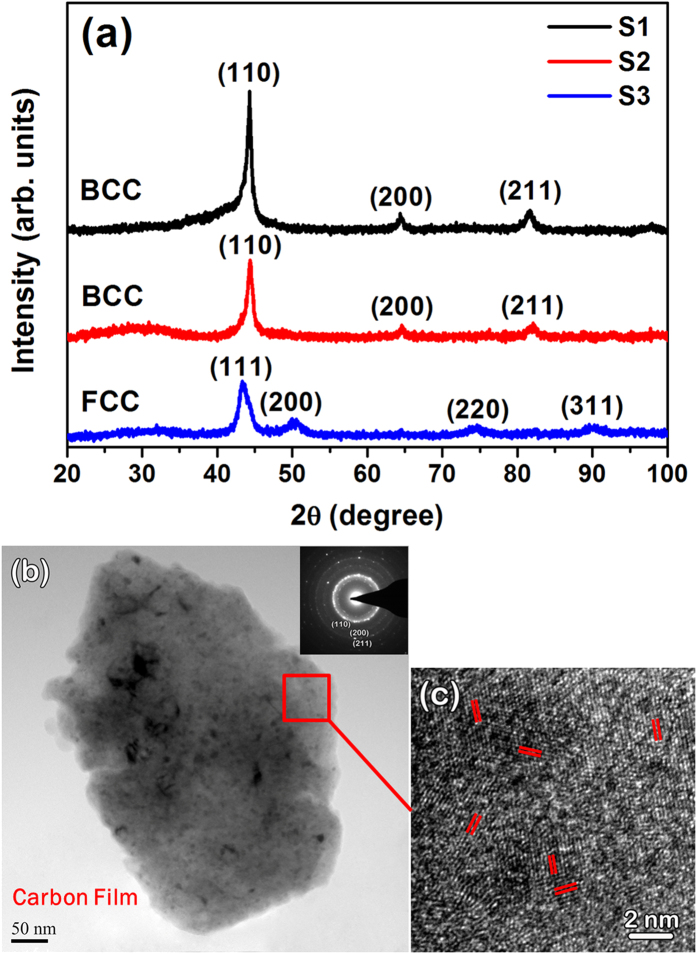
(**a**) XRD curves of the HEA AlCoCrTiZn (S1), AlCoCrFeNi (S2), and CoCrFeMnNi (S3) after 60 h milling. (**b**) TEM bright field image of theS1 particles after 60 h milling, andthe inset shows the corresponding selected area electron diffractionpattern. (**c**) Thecorresponding HRTEM imageobtained from the area marked in (**b**).

**Figure 2:**
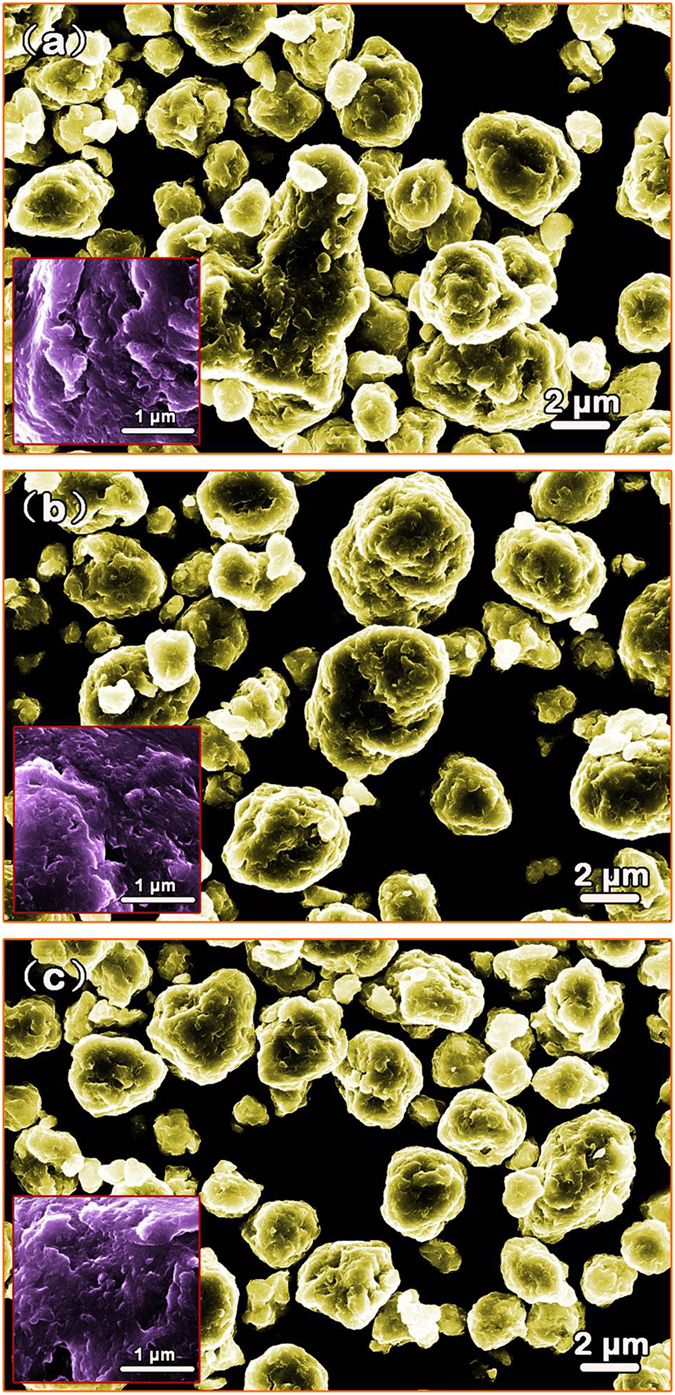
SEM secondary electron images of (**a**) S1, (**b**) S2 and (**c**) S3 after 60 h milling. The inset in each image shows the feature of the corresponding surfaces.

**Figure 3:**
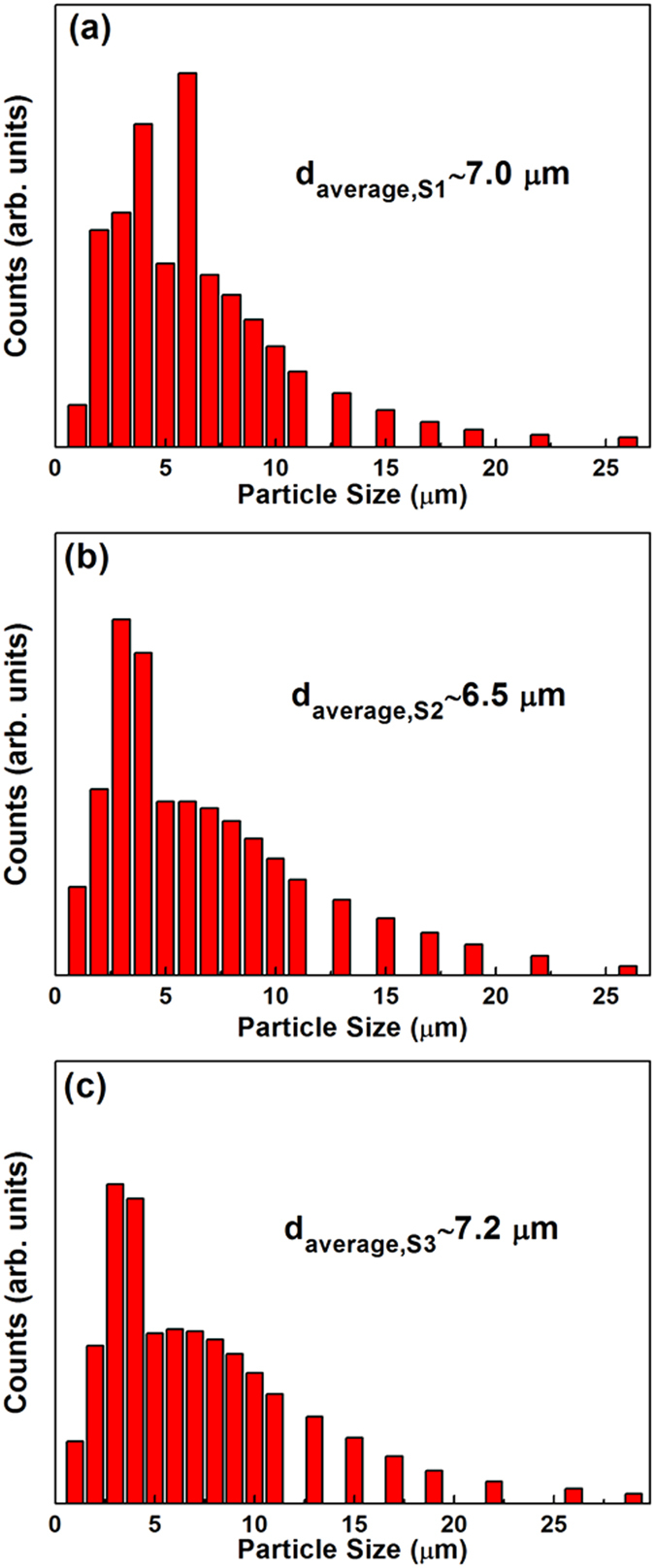
Size distribution of different HEA powders after being milled 60 h. (**a**) S1, (**b**) S2 and (**c**) S3.

X-ray diffraction (XRD) patterns of S1, S2 and S3 after milling for 60 h are shown in Fig. 1a. It is seen that the S1 and S2 powders have a BCC structure while the S3 powder shows a FCC structure. The BCC structure of S1 after milling for 60 h was further verified by the transmission election microscopy (TEM) bright field image (Fig. 1b) and its corresponding selected area electron diffraction (SAED) pattern (the inset in Fig. 1b). The crystalline feature of S1 was also clearly confirmed by high resolution TEM (HRTEM) observation (Fig. 1c), in which one can find obvious lattice fringes (highlighted with red lines in Fig. 1c) and defects attributed to the long-time ball milling. The scanning election microscopy (SEM) images of the S1, S2 and S3 powders after milling for 60 h are shown in Fig. 2a–2c, respectively. It is obvious that all the three powders are uniformly dispersed and have irregular profiles. The surfaces are rough and full of corrugations (see the corresponding insets in Fig. 2), which have significant effects on the degrading performance as will be discussed later on. The size distribution of the powders is presented in Fig. 3, and as shown, all the three HEA powders have a rather narrow size distribution from 1.0 to 13.0 μm. The statistic average size is 7.0, 6.5 and 7.2 μm in diameter for S1 (Fig. 3a), S2 (Fig. 3b) and S3 (Fig. 3b), respectively. The corresponding EDX analyses (the atomic percentages of Al, Co, Cr, Ti and Zn are 21.49%, 19.60%, 18.63%, 19.78% and 20.50%, respectively) of the powders verify that the actual composition of S1, S2 and S3 is close to the nominal. The specific surface area of the three samples was measured to be 3.635, 3.341 and 3.462 m^2^/g by the BET analysis with nitrogen for S1, S2 and S3, respectively.

### The azo dye degradation capacity of the HEA powders

**Figure 4:**
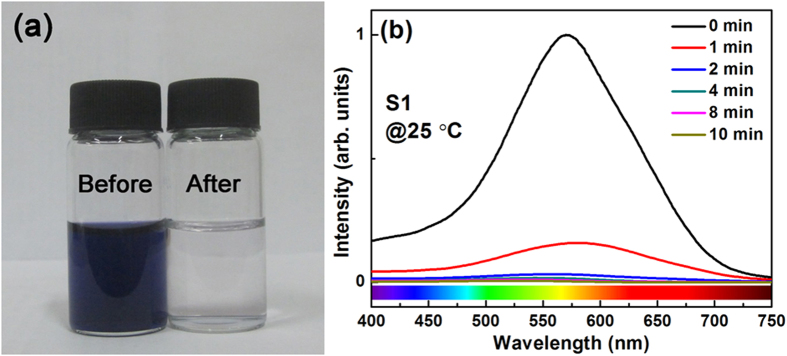
(**a**) Appearances of the DB6 solution before and after degradation by S1, and (**b**) changes of the UV absorption spectra of S1 measured at 25 °C as a function of the treating time.

**Figure 5:**
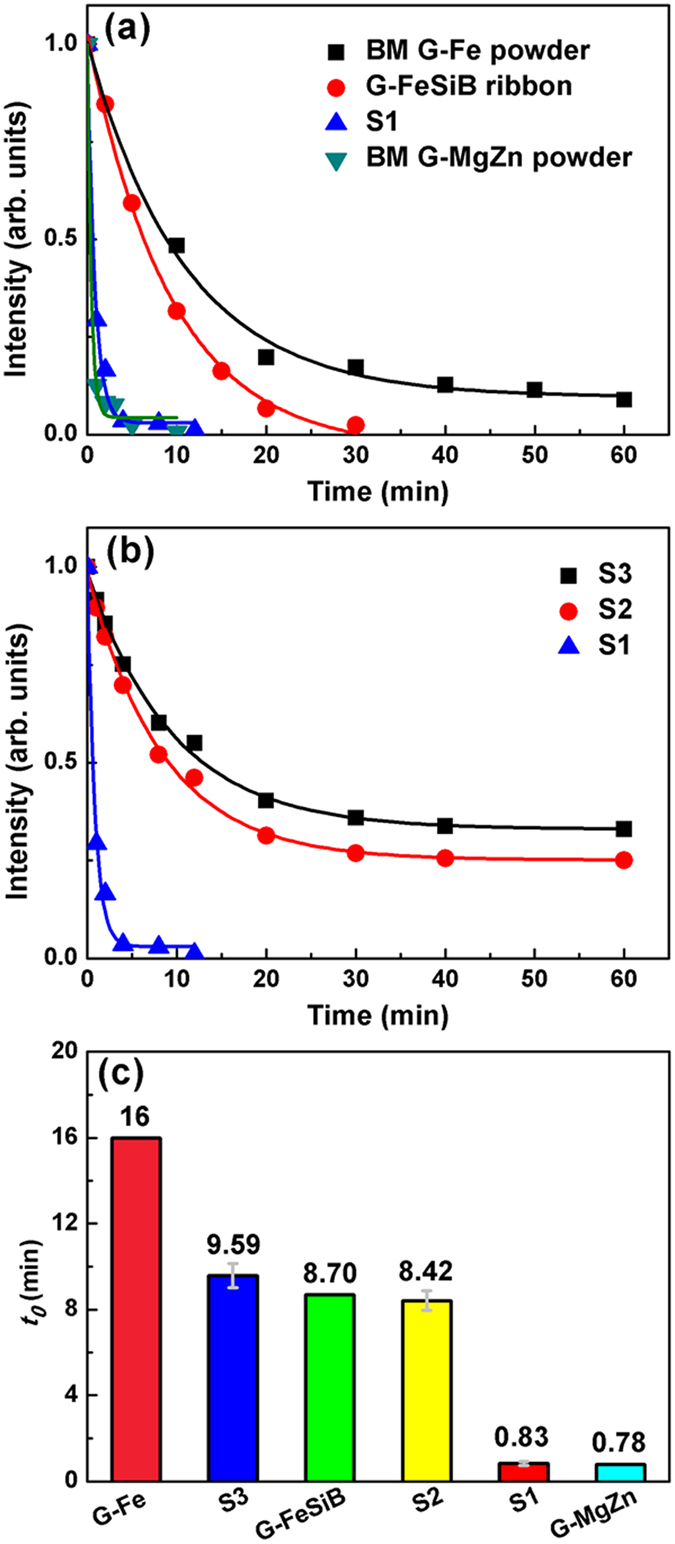
The normalized concentration as a function of degrading time at 25 °C (**a**) for BM G-Fe powder^19^, G-FeSiB ribbon^21^, S1 and BM G-MgZn^19^, and (**b**) for S3, S2 and S1. The solid lines are fitting curves by an exponential decay function. (**c**) Comparison of the reaction efficiency among different powders. Here, the concentration of all the DB6 solutions is 0.2 g/L while the dosage is different for different agents, i.e., 0.01 g/mL for HEAs powders, 0.0133 g/mL for G-FeSiB, 0.0134 g/mL for G-Fe, and 0.006 g/mL for G-MgZn.

To test the degradation capacity of the resultant HEAs, we put 0.1 g HEA powders into 10 ml azo dye solution with a concentration of 0.2 g/L Direct Blue 6 (DB6, C_32_H_20_N_6_S_4_O_14_Na_4_) each time. The representative appearance of the azo dye solution before and after degradation by HEA powders is shown in Fig. 4a. It is clear that the opaque blue solution becomes transparent, colorless water after degradation for 10 min. To quantitatively characterize the degrading kinetic behavior, we measured the concentration change of DB6 as a function of degrading time by the UV absorption spectrum. All the measured UV absorption spectra evolution with the degrading time exhibit a similar behavior. As an example, Fig. 4b shows a representative absorption curve for S1, and a diffuse peak was observed around 580 nm, corresponding to the wavelength scale of green and yellow colors, caused by the azo bond “-N=N-”. As the degradation reaction proceeds, the azo bond “-N=N-” breaks into two “-NH_2_”, which is often observed in degrading azo dyes by zero-valent iron^14^ and metallic glasses^21^. With the increase of the reaction time, an appreciable decrease in the intensity of the absorption peak, which is proportional to the concentration of azo dye solution, can be observed. The degradation occurred at a quite fast rate during the first minute, accompanied by a sharp drop of the absorption peak intensity. The azo dye was completely degraded without any detectable characteristic peaks after 10 min at 25 °C (Fig. 4b). In light of the quantitative UV absorption spectrum data, the degrading kinetic curve of HEA powders is plotted (Fig. 5), in comparison with that of other typical candidate materials. Apparently, the degradation rate of the S1 HEA powder at room temperature (25 °C) is faster than that of the Fe-Si-B amorphous alloy ribbon^21^ and the ball-milled (BM) Fe-based metallic glass powders^19^, and is even comparable with that of BM MgZn-based metallic glass powders^19^ (Fig. 5a). It is worth noting that the dosage of agents in our case is lower than that reported in the literature while the azo dye solution possesses the same concentration of 0.2 g/L DB6^19^,^21^. As comparison, Fig. 5b shows the degradation efficiencies for the S2 and S3 powders at 25 °C. In order to compare the degradation capability at 25 °C of different materials quantitatively, the normalized intensity of the DB6 concentration as a function of reaction time is plotted, which can be well fitted by an exponential decay function based on the pseudo-first-order kinetic model (solid lines in Fig. 5a-5b), *I* = *I*_0_ + *I*_1_ exp(−*t*/*t*_0_)^16^, where *I* is the normalized intensity of concentration, *I*_0_ and *I*_1_ are fitting constants, *t* is the reaction time and *t*_0_ is the time when the intensity decrease to e^−1^ of the initial condition. The degradation efficiency can be simply evaluated by the value of *t*_0_. Generally, HEAs are strong competitors of metallic glasses in the degradation efficiency (Fig. 5c). In particular, the efficiency of S1 (*t*_0_ = 0.83 min) is outstanding and fairly close to that of the BM MgZn-based glassy powder (BM G-MgZn, *t*_0_ = 0.78 min)^19^, which is the best one reported in the metallic glasses so far. It was reported that the BM G-MgZn is about 20 times faster than the BM Fe-based glassy powder (BM G-Fe, *t*_0_ = 16 min) and 1000 times faster than the gas-atomization Fe-based glassy powder (GA G-Fe, *t*_0_ = 810 min) in degradation of DB6^17^. It should be pointed out that the degradation efficiency of the BM G-Fe is about 200 times faster than that of the widely used commercial Fe powder^17^. Among the three HEAs, one can see that the newly developed S1 is the best (Fig. 5c) and its efficiency (*t*_0_ = 0.83 min) is more than 10 times faster than that of S2 (*t*_0_ = 8.42 min), S3 (*t*_0_ = 9.59 min) and the G-FeSiB ribbon (*t*_0_ = 8.70 min)^21^. Therefore, we will focus on the S1 for further analyses in the next section.

### Effect of temperature on degradation efficiency

**Figure 6:**
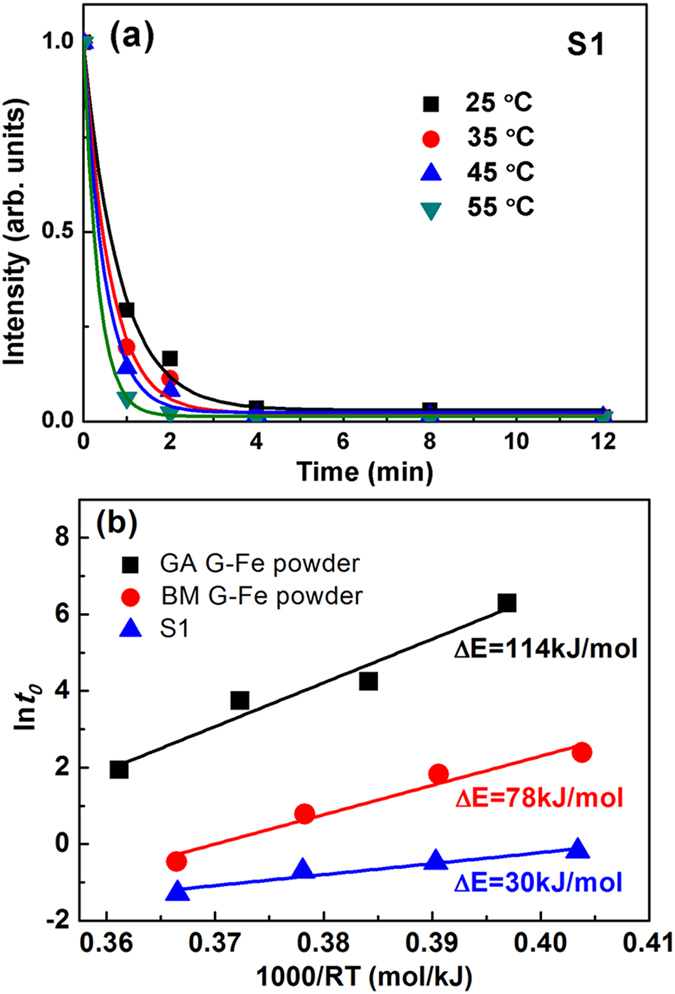
(**a**) The normalized concentration as a function of treatment time at different temperatures ranging from 25 to 55 °C. The solid lines are fitted by an exponential decay function. (**b**) Plot of (ln*t*_0_) vs. (1000/RT) for estimation of the degradation activation energy for GA G-Fe powder^17^, BM G-Fe powder^17^ and S1.

It is known that temperature has a significant effect on the degradation rate. To understand the temperature effect in our case, we performed a series of degradation reactions under various temperatures ranging from 25 to 55 °C with an interval of 10 °C. Figure 6a shows the variation of reaction efficiency with temperature for S1. Clearly, the higher the working temperature, the faster the degradation rate. Specifically, the *t*_0_ value drops from 0.83 at 25 °C to 0.34 at 55 °C. In addition, we estimated the thermal activation energy barrier Δ*E* with the Arrhenius-type equation, *t*_0_ = *τ_o_* exp(Δ*E*/*RT*), where *τ*_0_ is a time pre-factor and *R* the gas constant^38^. By plotting ln*t*_0_ versus 1000/*RT* curves, we obtained Δ*E* for the S1 and Fe powders synthesized by different techniques (Fig. 6b). The estimated Δ*E* value for S1 is 30 kJ/mol, which is only half of the BM G-Fe powder (78 kJ/mol^17^) and quarter of the GA G-Fe powder (114 kJ/mol^17^). The Δ*E* value of S1 is comparable with that of the BM G-MgZn powder, i.e., 51 ± 23 kJ/mol^19^. These results indicate that the newly developed S1 HEA powder can reduce the energy barrier for the degradation reaction effectively and thus make the occurrence of reaction easier as compared with other materials such as the glassy Fe-based powders.

## Discussion

**Figure 7:**
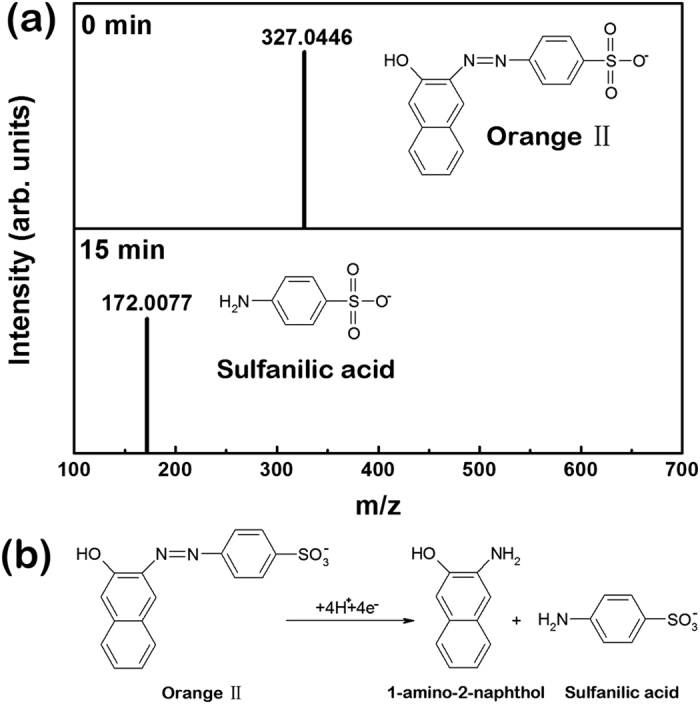
The negative mode HRMS of Orange II with a concentration of 0.2 g/L degraded by S1 (dosage of 0.01 g/ml) for 0 and 15 min at room temperature, respectively (25 °C).

As described above, the HEA powders have excellent performance on the degradation of azo dyes. In particular, S1 (i.e., the AlCoCrTiZn HEA) possesses superior degradation efficiency and good catalytic activity, which is much better than the commonly used zero-valent iron and recently developed BM Fe-based metallic glass powders. To further understand the underlying degradation mechanism, the intermediate products and the pathway of the reduction of azo dyes by S1 were also identified by high resolution mass spectra (HRMS). Similar to previous work^14^,^15^, Orange II was chosen as the original azo dye because of its simple structure which will provide convenience for HRMS testing. Figure 7a shows the HRMS result of Orange II with a concentration of 0.2 g/L degraded by S1 (dosage of 0.01 g/ml) for 15 min. As can be seen, Orange II was confirmed by the negative mode before degradation but after 15 min, Orange II completely degraded and only the sulfanilic acid was detected. From this test, it is known that the first step of the degradation of Orange II is the breaks of azo bond “-N=N-”, which is consistent with literature data^14^,^15^. In this case, sulfanilic acid and 1-amino-2-naphthol are supposed to be the possible intermediates. However, 1-amino-2-naphthol does not appear in Fig. 7a due probably to its auto-oxidation^21^. The above HRMS result confirms that the degradation of the azo dyes by S1 is similar to the reaction catalyzed by either crystalline iron powders or metallic glasses, namely, S1 provides electrons for the electron-deficient azo bonds, as the water providing the hydrogen, to disassemble the azo dyes into two parts. The reaction equation can be described by the expression shown in Fig. 7b.

The promising performance of our HEA powders in degrading contaminants is attributed mainly to the following three factors. The first one is the unique atomic structure of HEAs. Although HEAs were often characterized globally by simple solid solution phases, such as single FCC or BCC, their atomic arrangements are actually complex by nature due to their random or quasi-random atomic occupancies of the multiple principal elements. In fact, a precise description on the atomic occupancies of each constituent in HEAs remains a scientific challenge in the field. Currently, it is generally accepted that the lattice of HEAs is severely distorted due to the distinct differences of the atomic sizes among the constituent elements and the disordered atomic arrangements^33^,^35^. Apparently, the severe lattice distortion gives rise to nontrivial atomic level stresses and eventually makes atoms locate in thermodynamically non-equilibrium sites of the lattice^39^. We calculated the intrinsic residual strain with the method proposed by Ye *et al*.^40^. The result of calculation indicates that the root-mean-square (R.M.S.) residual strain of the excellent AlCoCrTiZn HEA is 4.92%, while the value for the CoCrFeMnNi and AlCoCrFeNi HEAs are 3.25% and 2.96%, respectively. As claimed by Ye *et al*., the transition from a single phase solid solution to multi-phased structure takes place at the R.M.S. residual strain of ~5%. The R.M.S. residual strain of AlCoCrTiZn, i.e., 4.92%, resides on the upper bound of the range of single phase solid solution. Meanwhile, we also calculated the delta-parameters with the method proposed by Zhang *et al*.^41^ to estimate the intrinsic lattice distortion of AlCoCrTiZn, CoCrFeMnNi and AlCoCrFeNi, which are 4.67%, 4.74% and 2.74%, respectively. As reported by Zhang *et al*.^41^, the solid solution structure is correlated with the delta-parameter of ~6%. The value of delta-parameter of AlCoCrTiZn, i.e. 4.67%, also locates at the upper bound of the range of single phase solid solution. In other words, the unique solid solution structure of HEAs virtually is in a non-equilibrium state where atoms possess high potential energy and are thus much more active than those in conventional alloys. As a result, the HEA needs a less energy to activate the reaction with the DB6 with respect to other materials. This point can be verified by the activation energy data. As shown in Fig. 6b, the activation energy barrier Δ*E* for the HEA (e.g., S1) is only half of the BM G-Fe powder and a quarter of the GA G-Fe powder, demonstrating that the HEA has a higher reactivity during degradation than the glassy Fe-based powders. This is the structural reason why the HEA exhibits a high degradation efficiency (Fig. 5c).

The second factor to affect the degradation properties of HEAs is the specific chemical composition. The chemical characteristics, especially the activity, of the constituent elements are also closely associated with the degradation performances of the resultant alloy. For instance, compared with S2 and S3 HEAs, S1 HEA exhibits a much better degradation capability, although they all have single-phase solid solution structures. This result can be qualitatively understood in terms of the activity of constituent metals. According to the activity series of metals^42^, the activity rank of the constituent elements in the studied HEAs is Al > Ti > Mn > Zn > Cr > Fe > Co > Ni. By comparing their chemical constituents of the HEA powders, one can find out that the constituents in the S1 HEA (AlCoCrTiZn) are of relative higher activity than those in the S2 (AlCoCrFeNi) and S3 (CoCrFeMnNi) HEAs. The degradation of azo dyes is essentially corresponding to the reduction of azo dyes. In the present case, it is the reduction of the azo bond “-N=N-” into two “-NH_2_” by the reducing agents, i.e., the HEA powders. The higher reactivity of constituents, the stronger reducing agents, leading to the faster reaction kinetics. Therefore, it is reasonable to infer that the chemical activity of constituent elements should be one of the decisive causes to the high degradation efficiency of the S1. Moreover, it is expected that the degradation efficiency of HEAs can be improved further by designing HEAs bearing elements with higher activities, such as Mg or Ca. However, it is kind of risky to synthesize HEAs containing high activity elements by the regular ball-milling due to the possibility of explosion at elevated temperatures. Thus, it is necessary to exploit a low-temperature ball-milling machine which is usually cooled by liquid nitrogen to produce HEA powders bearing Mg or Ca. The further investigation is in progress.

**Figure 8:**
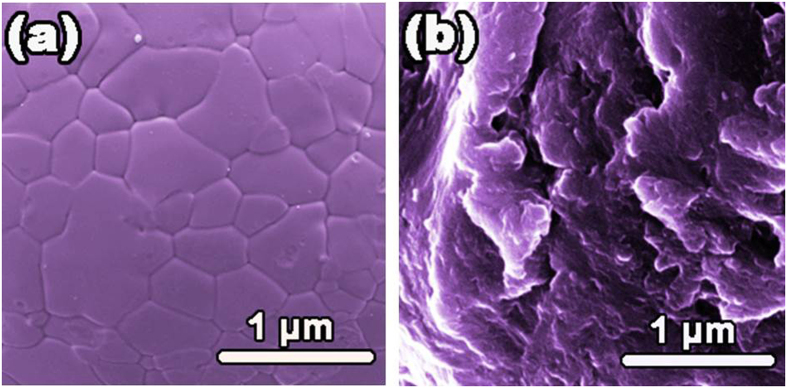
SEM images of the surface profile of the GA powder (**a**) and the 60 h milled powder (**b**) of S3.

Thirdly, the ball milling process plays a nontrivial role in affecting the degradation performance. It is known that ball milling process, especially for the long-time ball milling, could lead to severe plastic deformation and high residual stresses, which can push the atoms to the saddle points of the potential energy landscape and therefore can reduce activation energy barrier effectively. As evidenced by Wang *et al*.^17^, for the glassy Fe-based powders with a same composition, the activation energy for the BM powders is about 40 kJ/mol lower than that for the gas atomized powders. Moreover, the ball milling process makes the surface of particles rough and full of corrugations (Fig. 2), leading to the significant increase of the specific surface area. To illustrate this point, we measured the specific surface areas for two kinds of HEA powders, which have the same composition of S3 and similar average particle size (~17 μm) while they were prepared respectively by BM and GA techniques. The results show that the specific surface area is 1.722 and 1.124 m^2^/g for the BM and the GA powders, respectively, verifying the significant increase in specific surface area for the sample after the BM process. To further demonstrate this result intuitively, we compared the surface profile of the GA powders with that of the BM powders. As shown in Fig. 8, the GA powder has a smooth surface (Fig. 8a) while the BM powder possesses a corrugated one (Fig. 8b), which renders the high specific surface area of the BM powders. It is known that high specific surface area is beneficial for the reaction rate and/or capacity due to more active spots generated. Consequently, the ball milling process has a substantial contribution to the excellent performance of the HEAs on degrading azo dye. Actually, similar results were also reported in the BM G-Fe powder^17^.

In addition, it is noticed that surface chemical segregation has a significant effect on surface activity, and is critical for degradation performance. In fact, surface segregation has been widely investigated for various alloys, especially bimetallic nanoparticles, applied in the field of catalyst engineering^43^,^44^. For example, Janik-Czachor *et al*. modified the surface activity of Cu-based amorphous alloys by making a selective surface segregation of Cu with chemical processes, leading to a substantial increase in the catalytic efficiency of dehydrogenation of aliphatic alcohols^43^. In our case, however, surface chemical segregation of the as-milled HEA powders should be subtle due to the fact that mechanical alloying is capable for homogenizing chemical elements^23^. In fact, our EDX analyses for different sites of the HEA particles indicate that there is no obvious chemical heterogeneity. As such, effects of surface chemical segregation on the degradation performance can be negligible in the case of as-milled HEA powders. On the other hand, the ball milling process renders the samples abundant of corrugations and defects on the particle surfaces, as shown in Figs 1b, 2 and 8b, which enhance the surface activity of the HEA powers effectively by providing a great deal of surface active spots for degradation reactions. In this regard, therefore, it is reasonable to infer that the good performance of S1-AlCoCrTiZn HEA is closely related to its high surface activity.

It is critical for the engineering application of HEAs in degrading azo dyes to clarify the concern whether the HEA powders generate secondary pollution or not during the degradation of azo dye solutions. To address this issue, we utilized the ICP (inductive coupled plasma) technique to detect the concentrations of metal ions in the treated solution. The results indicate that the ion concentrations of all the five constituents in the treated solutions are below their detection limits of the machine, i.e., 0.067, 0.016, 0.017, 0.005 and 0.012 μg/ml for Al, Co, Cr, Ti and Zn, respectively, clearly demonstrating that the degradation of azo dyes by using HEA powders as agents does not induce secondary pollution.

In summary, we demonstrated that the HEA powders synthesized by mechanical alloying, particularly the newly developed AlCoCrTiZn HEA, exhibited high efficiency in degradation of azo dye, comparable with the best metallic glasses. The combination of unique atomic structure with severe lattice distortion, chemical composition effect, residual stress and high specific surface area is responsible for the observed excellent capability of HEAs in degrading azo dye. Our findings manifest that the newly developed HEAs has great potential to be utilized as catalyst materials such as purifying wastewater, and the current study broadens application ranges of HEAs.

## Methods

### Synthesis of high entropy alloy powders

Al, Ti, Cr, Mn, Fe, Co, Ni, Zn elemental powders with high purity (>99.5%) and particle size of less than 45 μm were used as raw materials to synthesize equal-atomic AlCoCrTiZn, AlCoCrFeNi and CoCrFeMnNi HEAs by mechanical alloying. The milling was carried out in high energy planetary ball miller at 300 rpm with a ball to powder ratio of 15:1. Stainless steel vials and balls were used as the milling media and n-heptane was utilized as the process controlling agent (PCA) in order to keep off from excessive cold welding. In addition, high purity argon gas was used for protection. The powder samples were extracted at a certain interval of 5 h to confirm the alloy formation. After 60 h of milling the powders were completely taken out for further characterization and testing.

### Reaction process

The azo dye, direct blue 6 (i.e., DB6, C_32_H_20_N_6_S_4_O_14_Na_4_, CAS 2602-46-2) and Orange II (C_16_H_11_N_2_SO_4_Na, CAS 633-96-5) were purchased from Hailan Chemical Pigment Co. (Tianjin, China). Deionized water was used as the solvent throughout the experiment. The concentration of both DB6 and Orange II solutions used was 0.2 g/L in this work. For all the degradation tests, 0.1 g HEA powders were dissolved into 10 ml of azo dyes solution for reaction. At preset reaction time intervals, about 5 ml solution was removed and filtered to measure the ultraviolet-visible (UV) absorption spectrum.

### Characterization

Atomic structure of the milled samples was studied by X-ray diffractometry (XRD) (MXP21VAHF, Cu*K*_α_) and transmission electron microscopy (TEM) (JEM-2010). The TEM samples were prepared by dropping powders dispersed in absolute ethanol upon copper grids with carbon film as voids support. After the absolute ethanol completely volatilized, the samples then be investigated by TEM. The morphology and size distribution were studied by SEM (Zeiss Supra 55), and the compositions of the milled powders were examined by EDX equipped on the SEM. The statistic distribution of size was obtained by laser particle analyzer (Mastersizer 2000). Thermal analysis was carried out in a differential scanning calorimeter (NETZSCH DSC 404F1). The Brunauer-Emmett-Teller (BET) surface area analysis of the powders was performed using the nitrogen adsorption method with a surface analyser (NOVA4000). The UV absorption spectrums were recorded using an UV spectrometer (Unico 2800). The concentrations of metal ions left behind the treatment solution were measured by ICP emission spectrometer (Leeman PROFILE SPEC). The intermediate products and the pathway of the reduction of azo dyes by S1 were identified by high resolution mass spectra (HRMS, LCMS-IT/TOF Shimadzu Japan).

## Additional Information

**How to cite this article**: Lv, Z. Y. *et al*. Development of a novel high-entropy alloy with eminent efficiency of degrading azo dye solutions. *Sci. Rep.* **6**, 34213; doi: 10.1038/srep34213 (2016).
